# Epstein-Barr virus activates F-box protein FBXO2 to limit viral infectivity by targeting glycoprotein B for degradation

**DOI:** 10.1371/journal.ppat.1007208

**Published:** 2018-07-27

**Authors:** Hao-Jiong Zhang, Jinxiu Tian, Xue-Kang Qi, Tong Xiang, Gui-Ping He, Hua Zhang, Xibao Yu, Xiao Zhang, Bingchun Zhao, Qi-Sheng Feng, Ming-Yuan Chen, Mu-Sheng Zeng, Yi-Xin Zeng, Lin Feng

**Affiliations:** 1 Department of Experimental Research, Sun Yat-sen University Cancer Center, State Key Laboratory Oncology in South China, Collaborative Innovation Center for Cancer Medicine, Guangzhou, China; 2 Department of Nasopharyngeal Carcinoma, Sun Yat-sen University Cancer Center, Guangzhou, P. R. China; Tulane Health Sciences Center, UNITED STATES

## Abstract

Epstein-Barr virus (EBV) is a human cancer-related virus closely associated with lymphoid and epithelial malignancies, and EBV glycoprotein B (gB) plays an essential role in viral entry into both B cells and epithelial cells by promoting cell-cell fusion. EBV gB is exclusively modified with high-mannose-linked *N*-glycans and primarily localizes to the endoplasmic reticulum (ER) with low levels on the plasma membrane (PM). However, the mechanism through which gB is regulated within host cells is largely unknown. Here, we report the identification of F-box only protein 2 (FBXO2), an SCF ubiquitin ligase substrate adaptor that preferentially binds high-mannose glycans and attenuates EBV infectivity by targeting *N*-glycosylated gB for degradation. gB possesses seven *N*-glycosylation sites, and FBXO2 directly binds to these high-mannose moieties through its sugar-binding domain. The interaction promotes the degradation of glycosylated gB via the ubiquitin-proteasome pathway. Depletion of FBXO2 not only stabilizes gB but also promotes its transport from the ER to the PM, resulting in enhanced membrane fusion and viral entry. FBXO2 is expressed in epithelial cells but not B cells, and EBV infection up-regulates FBXO2 levels. In summary, our findings highlight the significance of high-mannose modification of gB and reveal a novel host defense mechanism involving glycoprotein homeostasis regulation.

## Author summary

EBV envelope glycoprotein B (gB) is a core component of the fusion machinery and plays an essential role in viral entry. EBV gB has been characterized as a high-mannose-containing glycoprotein, but the significance of this sugar type is unknown. In this work, we report the identification of FBXO2, a glycosylation-dependent E3 ubiquitin ligase component recognizing *N*-linked high-mannose oligosaccharides, as a novel gB-interacting host protein that controls the levels of gB glycosylation and its membrane transport. The homogenous high-mannose type *N*-glycan structure as a “script” that directs the degradation of the glycosylated gB by SCF^FBXO2^ E3 ubiquitin ligase. Our results propose a novel host defense mechanism by which host cells sense the penetrated EBV and attenuate the infectivity of progeny virus.

## Introduction

Epstein-Barr virus (EBV) belongs to the γ-herpesvirus family, and more than 90% of individuals worldwide are asymptomatically infected with EBV. As the first discovered tumor virus, EBV has been identified in Burkitt’s lymphoma, Hodgkin’s disease, a subset of gastric cancers and nearly all non-keratinizing nasopharyngeal carcinomas (NPCs), the dominant histological subtype of NPC in endemic regions, including southern China and Southeast Asia [1–3]. EBV is only capable of infecting human B lymphocytes and epithelia. EBV adopts two lifestyles: latent and lytic phases. The current model of the EBV lifecycle proposes that EBV is transmitted in saliva and crosses the oral mucosal epithelium to infect B lymphocytes, where it establishes a lifelong latent infection. EBV then occasionally switches to a lytic replication phase in epithelial cells for shedding virions to saliva or for infection of more B cells to replenish the virus reservoir [4].

The entry of EBV into host cells is a complex process mediated by multiple viral envelope glycoproteins. Glycoprotein B (gB), gH/gL, gp42 and gp350 are required for EBV entry. gp350 and gp42 determine cell tropism and function by receptor binding [5,6]. gB, gH and gL form the core viral fusogen complex and are essential for entry into all cell types. Besides, binding of gH/gL to integrin and ephrin receptor A2 has been proposed to be necessary for the infection of epithelial cells [7–9].

Viral fusogens mediate the fusion of the viral envelope and host membrane during entry and egress. It has been suggested that gB catalyzes fusion itself, whereas the heterodimer gH/gL plays a regulatory role. gB/gp110, encoded by the *BALF4* ORF in EBV, is expressed during the lytic phase [10]. gB is a type I single-pass membrane protein that exists as a trimer. It harbors a large N-terminal ectodomain, a transmembrane domain and a short C-terminal tail. Unlike gp350, gH/gL and gp42, which attach to host cells by binding to their respective receptors, gB exhibits inherent fusogenic properties. Structurally, herpesvirus gB adopts a similar “hairpin” conformation, including a trimeric fold and bipartite fusion loop [11], which led to the classification of herpesvirus gB as a class III viral fusogen [12]. Based on the available post-fusion crystal structure of EBV gB and the pre- and post-fusion conformations of herpes simplex virus type 1 (HSV-1) gB, it is proposed that gB undergoes dramatic prefusion to post-fusion conformation changes to insert fusion loops into target cell membranes and drive membrane fusion [13–16].

Despite the high conservation and structural similarities among herpesvirus gB [14,16], EBV gB exhibits some unique properties. For example, gB of α-herpesviruses, such as HSV-1 and HSV-2 gB, are very abundant envelope proteins on virions [17,18]. In contrast, EBV gB is predominantly localized in the endoplasmic reticulum (ER) [19] and exhibits low levels of cell surface expression and virion incorporation, therefore the virion abundance of gB is an important virulence factor for EBV infection [20]. The difference in subcellular distribution reflects the different glycan types on these gBs. Viral envelope glycoproteins are processed in the secretory compartment of host cells, where they are decorated with various types of oligosaccharides. In the ER, the protein is modified with high-mannose oligosaccharides consisting of Man_5-9_GlcNAc_2_ structures on an Asn residue; once the proteins traffic to the Golgi, high-mannose glycans are further modified by the addition of various sugar residues to form hybrid and complex *N*-glycans [21]. Therefore, the ER-retained EBV gB is only modified with high-mannose *N*-linked oligosaccharides [10,22–25], whereas HSV gBs are enriched in complex *N*-glycans [17,18]. Because viral fusion proteins usually exist on the viral membrane surface to promote membrane merging, how host cells limit the processing and maturation of gB remain elusive. In this study, we report the identification of FBXO2/Fbx2/Fbs1/OCP1, a glycan-dependent E3 ubiquitin ligase component recognizing *N*-linked high-mannose oligosaccharides [26–30], as a novel EBV gB-interacting protein that controls the level and membrane transportation of gB in epithelial cells to decrease the infectivity of progeny viruses.

## Results

### Identification of FBXO2 as a novel gB-interacting host protein

**Fig 1:**
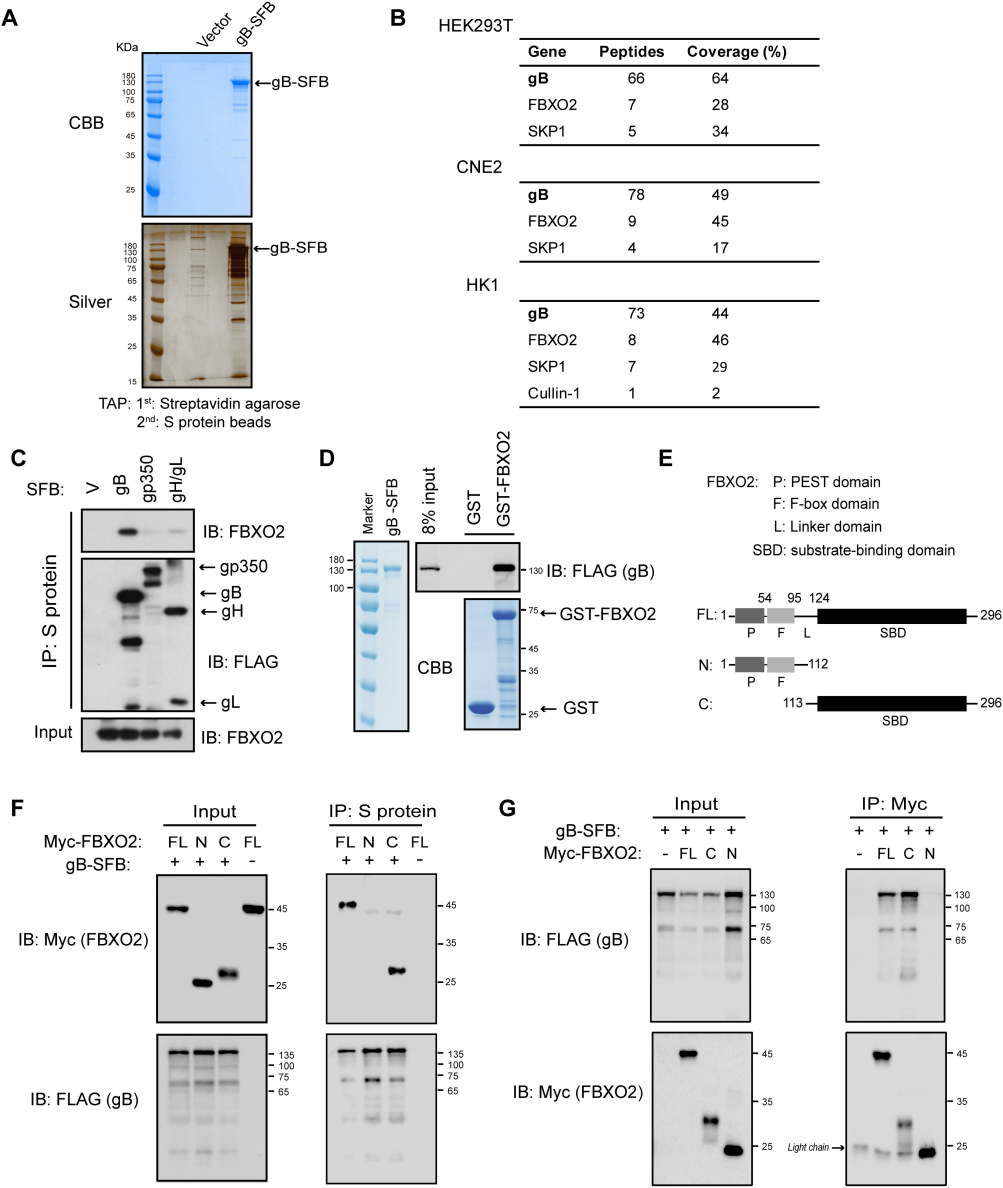
FBXO2 binds to gB via its substrate/sugar-binding domain. **(A)** TAP of C-terminal SFB-tagged gB in HEK293T cells. Final eluted proteins were analyzed by Coomassie Brilliant Blue (CBB; top) or silver staining (bottom). **(B)** Summary of proteins identified by TAP-MS analysis in HEK293T, CNE2 and HK1 cell lines stably expressing gB-SFB. **(C)** HEK293T cells were transfected with plasmids encoding SFB-tagged gB, gp350, gH/gL or empty vector. Cell lysates were subjected to precipitation using S-protein beads, and the bound protein was determined by Western blotting (WB). **(D)** Direct binding of gB to FBXO2. Bacterially purified GST-FBXO2 or GST alone was incubated with gB purified from stably infected HEK293T cells. Proteins bound to glutathione Sepharose beads were analyzed by WB. **(E)** Schematic representation of the FBXO2 domain structure. P, PEST domain; F, F-box domain; SBD, substrate-binding domain; FL, full-length. **(F)** Co-IP of FBXO2 fragments and gB. HEK293T cells were co-transfected with plasmids encoding Myc-tagged FL FBXO2 or N- or C-terminal FBXO2 fragments and gB-SFB. Cell lysates were precipitated with S-protein beads and immunoblotted with the indicated antibodies. **(G)** Reciprocal co-IP of gB and FBXO2 fragments. The procedures were similar to those performed in **(E)** except that the cell lysates were immunoprecipitated with anti-Myc agarose. The arrowhead indicates IgG light chain. The sizes of molecular mass markers (M) are shown in kDa.

To better understand the interplay between EBV gB and host proteins, tandem affinity purification (TAP) [31] of gB was performed in human cells. Following two-step purification of triple-tagged (S-Flag-Streptavidin-binding peptide; SFB) gB in HEK293T cells and two NPC cell lines, CNE2 and HK1, proteins associated with gB were identified by mass spectrometry (MS) analysis (Fig 1A). Interestingly, a significant number of peptides corresponding to an F-box protein named FBXO2, also known as FBS1/FBX2/NFB42/OCP1, were repeatedly identified in the TAP-MS data of the three cell lines stably expressing gB. FBXO2 is the substrate recognition component of the SCF (SKP1/Cul1/F-box protein) E3 ubiquitin ligase complex, and accordingly, SKP1 and Cullin-1 were also identified in gB immunocomplexes (Fig 1B) (see S1–S3 Tables for the complete TAP-MS data). The association with SCF^FBXO2^ seems to be specific to gB because TAP of other EBV glycoproteins, such as gp350, gH/gL and gp42, did not identify SCF^FBXO2^ polypeptides but did capture their respective receptors, including CR2, integrin and MHC-II (S1A Fig). Thus, our TAP approach was applicable for studying the host proteins interacting with EBV glycoproteins.

To verify the association between gB and FBXO2, we precipitated the ectopically expressed gB, gp350 and gH/gL from the transfected HEK293T cells and examined the presence of FBXO2 in the viral envelope protein immunocomplex. We found that only gB, but not gp350 and gH/gL, strongly interacts with FBXO2 (Fig 1C). Furthermore, reciprocal immunoprecipitation (IP) also confirmed the specific binding of gB to FBXO2, whereas another F-box protein, FBXO3, was unable to bind gB (S1B and S1C Fig). Next, we sought to determine whether the interaction is direct. A GST pull-down experiment was performed using recombinant GST-FBXO2 or GST protein derived from *E*. *coli* and gB protein derived from mammalian cells, and the data revealed a strong interaction between gB and FBXO2 (Fig 1D). As a substrate adaptor in the SCF complex, FBXO2 binds to SKP1 via an F-box domain and binds to substrates via the C-terminal substrate-binding domain, which is also termed the sugar-binding domain (SBD) because it recognizes sugar moieties on substrates [30]. To determine the region responsible for gB binding, two FBXO2 truncation mutants, FBXO2-N, which contains the PEST and F-box domains, and FBXO2-C which harbors the SBD domain, were generated (Fig 1E). Co-IP experiments demonstrated that gB only precipitated full-length FBXO2 and FBXO2 SBD but not FBXO2-N (Fig 1F), and reciprocal co-IP obtained similar results (Fig 1G). These data suggest that gB may represent a potential substrate of SCF^FBXO2^.

### FBXO2 is expressed in nasopharyngeal and oral epithelial cells but not in B cells and is up-regulated by EBV infection

**Fig 2:**
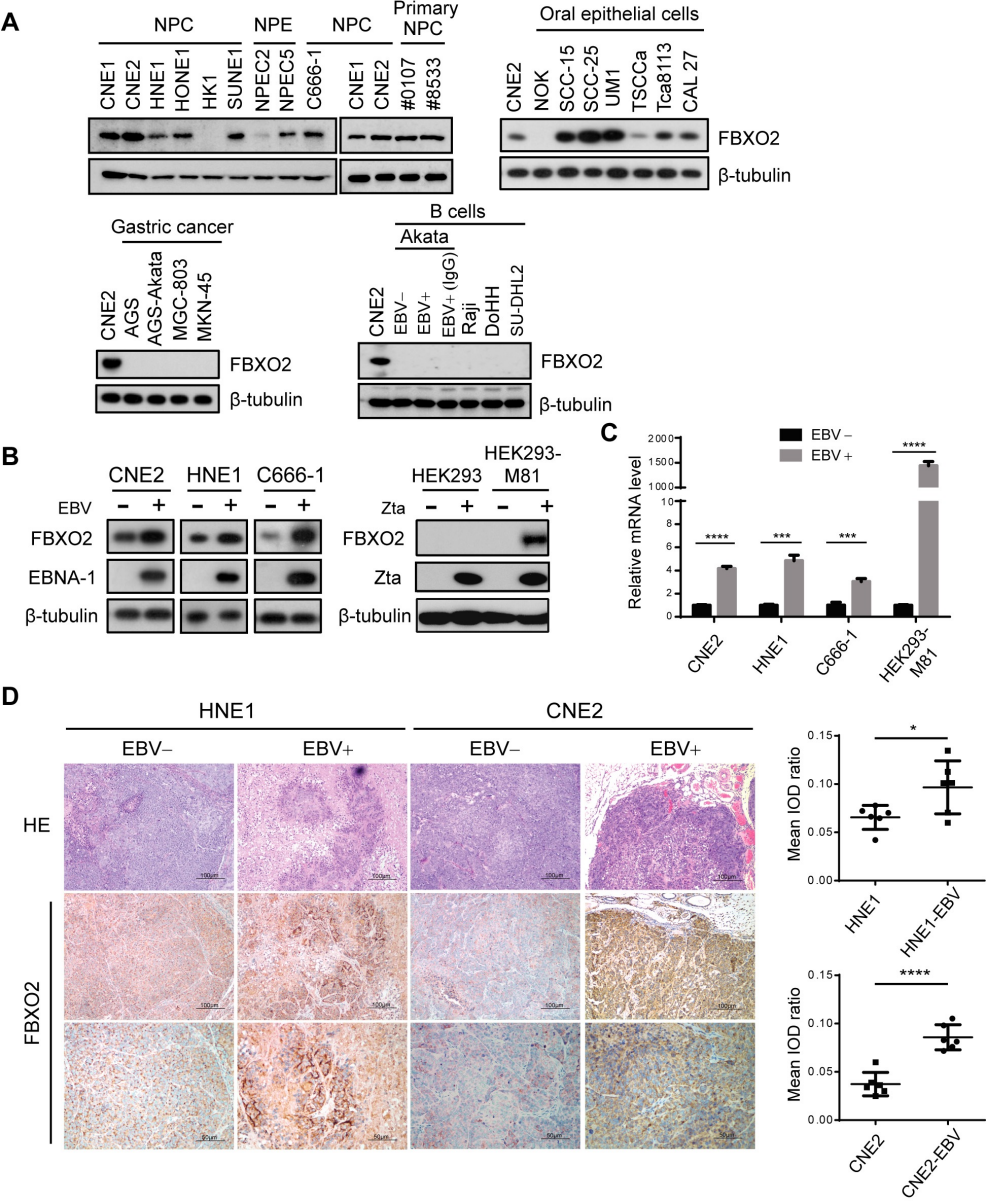
FBXO2 is expressed in NPC cells and is induced upon EBV infection. **(A)** Immunoblotting of FBXO2 in different cell types. Seven NPC cell lines, two primary NPC cell lines derived from NPC patients (#0107 and #8533 are the IDs of the patients enrolled in Sun Yat-sen University Cancer Center), two immortalized NPE cell lines, oral epithelial cell lines, gastric cancer cell lines and B cell lines were examined. Akata-EBV+ cells were treated with or without anti-human IgG for lytic induction. **(B)** (left) FBXO2 expression in paired EBV-positive and EBV-negative NPC cells. WB analysis was performed in HNE1 and CNE2 cells infected or not with EBV produced by Akata cells and in EBV-positive C666-1 NPC cells that were kept intact or subjected to CRISPR/Cas9-mediated *EBNA-1* deletion by two EBNA-1 gRNAs to abrogate EBV replication. (right) HEK293 control cells or cells carrying the M81/ΔZta EBV episome were transfected with a plasmid encoding Zta or empty vector, and the expression of FBXO2 and Zta was analyzed by immunoblotting. **(C)** Relative mRNA levels of FBXO2 determined by real-time PCR in cells as in **B**. n = 3. **(D)** (left) Representative images of H&E and IHC staining for FBXO2 in xenograft tumors derived from EBV-negative and EBV-positive NPC cells. Scale bars, 100 μm. (right) Tissue image analysis with Image-Pro Plus 6.0 according to the average integrated optical density (IOD) per stained area (μm^2^) (IOD/area). Twenty independent images taken from six xenografts in each group were analyzed, and the results are shown as the means ± SD. *P < 0.05; **P < 0.01, ***P < 0.001, ****P < 0.0001; Student’s t-test.

FBXO2 was originally described as a brain-specific F-box protein [32–34] and has also been identified in cochlear cells [35]; accordingly, FBXO2-knockout mice develop age-related hearing loss [36]. Recently, FBXO2 was reported to be up-regulated in the livers of obese mice, and the insulin receptor was identified as a substrate of FBXO2 [37]. Thus, whether FBXO2 is expressed in EBV host cells, including epithelial cells of the nasopharynx, oral cavity and stomach, and B lymphocytes, needs to be determined. Interestingly, cells originating from the nasopharynx epithelium, including six NPC cell lines, two primary NPC cell lines, and two immortalized nasopharyngeal epithelial (NPE) cell lines, all expressed considerable amounts of FBXO2, with the exception of HK1, which is the only well-differentiated squamous carcinoma cell line and is less representative for NPC [38]. Besides, FBXO2 was highly expressed in oral cancer cell lines but absent in normal oral keratinocytes (NOK). In contrast, FBXO2 was undetectable in four gastric cancer cell lines we examined, including EBV-positive AGS cell line, it might because of the different cancer types, as most gastric tumors are adenocarcinoma, while more than 90% of all oral cancers are squamous cell carcinoma, and the majority of NPCs are the undifferentiated carcinoma. On the other side, none of the B cell lines examined expressed FBXO2, including the EBV-negative non-Hodgkin's lymphoma B cell lines DoHH2 and SU-DHL-2, the EBV-positive Burkitt's lymphoma (BL) cell lines Raji and Akata and an EBV-negative Akata cell line, and the induction of EBV into lytic replication by IgG crosslinking did not induce FBXO2 expression in Akata-EBV+ cells (Fig 2A). We next examined the FBXO2 levels in paired EBV-negative and EBV-positive NPC cell lines. Two typical NPC cell lines, CNE2 and HNE1, were infected with recombinant EBV produced in Akata cells. C666-1, the only native EBV-infected NPC cell line, was subjected to EBV genome destruction by CRISPR/Cas9-mediated *EBNA-1* deletion as described previously [39]. Intriguingly, EBV infection profoundly increased FBXO2 protein levels in the three pairs (Fig 2B, left). Similarly, activating EBV production by transfecting Zta into HEK293 producer cells carrying a recombinant M81/ΔZta episome [40] substantially up-regulated FBXO2 expression (Fig 2B, right). Quantitative PCR revealed that all these EBV-positive cells had higher FBXO2 mRNA levels than their virus-free counterparts (Fig 2C), suggesting that EBV infection stimulates FBXO2 transcription in NPC and HEK293 cells. Furthermore, immunohistochemistry (IHC) of xenograft tumors derived from EBV-infected and uninfected NPC cells also support the positive correlation between EBV status and FBXO2 expression (Fig 2D).

### The interaction of gB with FBXO2 is dependent on high-mannose-type *N*-glycosylation of gB

**Fig 3:**
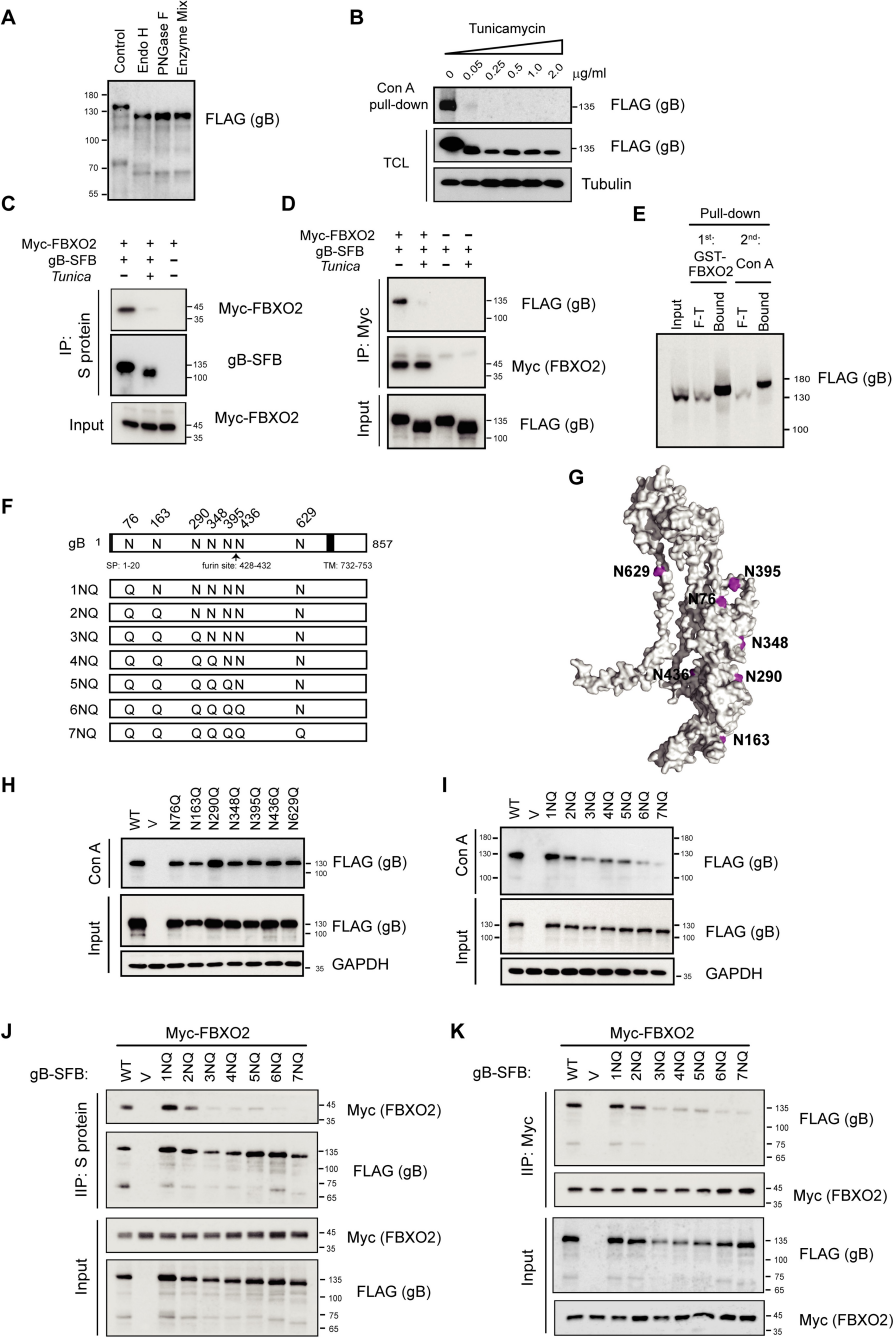
*N*-linked high-mannose glycosylation of EBV gB is required for FBXO2 interaction. **(A)** Analysis of glycan modifications of gB. gB-SFB purified from CNE2 cells stably expressing the protein was treated with endoglycosidase H (Endo H) or PNGase H or a Deglycosylation Mix before electrophoresis on 8% polyacrylamide gels. The sizes of molecular mass markers (M) are shown in kDa. **(B)** CNE2 cells stably expressing gB-SFB were treated with tunicamycin at different concentrations as indicated overnight before harvest. Con A agarose was used to enrich *N*-linked glycoproteins. TCL: total cell lysates. **(C-D)** Inhibition of N-linked glycosylation of gB by tunicamycin abrogates the gB-FBXO2 association. HEK293T cells transfected with plasmids encoding Myc-FBXO2 and gB-SFB were treated with 0.25 μg/mL tunicamycin or DMSO for 24 h, and the cells were harvested and subjected to immunoprecipitation by S-protein agarose **(C)** or anti-Myc agarose **(D)**. **(E)** Sequential pull-down of glycosylated gB by GST-FBXO2 followed by Con A agarose pull-down. Eight percent of the TCL of cells stably expressing gB was loaded as input; F-T: 8% of the flow-through. **(F)** Schematic diagram of glycosylation sites on gB and its N-to-Q mutants. The signal peptide (SP), furin cleavage site and transmembrane (TM) domain of gB are indicated. **(G)** Ribbon diagrams of the monomeric EBV gB structure, illustrated with the PyMol program using PDB 3FVC as the template. The glycosylation sites as shown in **(F)** are colored in magenta. **(H-I)** Con A agarose pull-down of gB single **(H)** or multiple **(I)** N-to-Q mutants. **(J-K)** Co-IP of FBXO2 with glycosylation-defective gB mutants. HEK293T cells were co-transfected with Myc-FBXO2 and gB N-to-Q mutants as indicated, and the cell lysates were subjected to immunoprecipitation by S-protein agarose **(J)** or anti-Myc agarose **(K)** and immunoblotted with anti-Myc and anti-FLAG antibodies.

FBXO2 specifically binds high-mannose oligosaccharides through its SBD [30], and EBV gB has been characterized as a high-mannose-containing glycoprotein [10,22,25]. The EBV gB gene encodes a mature protein of 836 amino acids, equivalent to a molecular weight of 93 kDa. Its apparent molecular mass is 110 kDa. Therefore, gB has also been designated gp110. We first characterized the gB glycan type by biochemical analysis. Addition of an SFB tag (25 kDa) resulted in a 135-kDa recombinant protein. Digestion of the SFB-tagged gB with endoglycosidase H (Endo H), a glycosidase that cleaves only *N*-linked sugars containing more than three mannose moieties, removed a glycan mass of approximately 15 kDa from gB. Treatment with peptide N-glycosidase F (PNGase F), which cleaves all types of *N*-glycans, or a deglycosylation mix that cleaves both *N*- and *O*-linked glycans did not cause a further reduction in molecular weight over that induced by Endo H, indicating a lack of complex *N*-linked and *O*-linked oligosaccharides on gB (Fig 3A), consistent with previous reports [10,22]. We then treated gB-expressing cells with tunicamycin, a GlcNAc transferase inhibitor that blocks the first step of *N*-linked glycosylation. Western blotting analysis revealed that tunicamycin treatment resulted in a complete loss of glycosylated gB, as demonstrated by the reduction in molecular weight and abolishment of Concanavalin A (Con A) agarose binding. Con A is an α-mannose/α-glucose-binding lectin that binds high-mannose and hybrid *N*-glycans, but not complex *N*-glycans [41] (Fig 3B). Therefore, we used tunicamycin to obtain de-glycosylated gB in cells. Co-IP experiments suggested that only fully glycosylated gB, but not de-glycosylated gB, associated with FBXO2 (Fig 3C), and vice versa (Fig 3D). Furthermore, GST-FBXO2 captured most gB from the lysates of gB-expressing cells, and when the flow-through (F-T) was subjected to a second round of pull-down by Con A agarose, less gB was obtained and migrated slower than that enriched by FBXO2 (Fig 3E), suggesting that in addition to high-mannose glycans, hybrid *N*-glycans are also present on EBV gB, but to a much lesser extent. These results also indicated that FBXO2 exhibits a narrower binding range than Con A for *N*-glycans.

We next aimed to identify the N-linked glycosylation sites on gB. EBV gB has nine putative *N*-glycosylation sites (Asn-X-Ser/Thr; X denotes any amino acid except proline). To determine the glycosylation sites on gB, we purified ectopic gB from HEK293T cells and performed *N*-glycan MS analysis (Fig 3E). MS identified five glycosylation sites on gB at N76, N290, N348, N395 and N436 (S2 Fig). In addition, previous structural analysis of gB revealed that N-acetyl glucosamine (NAG) molecules could be found at positions N163, N290 and N629 [14]; thus, N163 and N629 were also included in our study (Fig 3F). According to the crystal structure of the EBV gB ectodomain [14], we modeled the locations of *N*-glycans. N163 and N290 lie in domain I, N76 and N348 lie in domain II, and N629 is located in domain V, and these glycan sites are distributed on the surface of the gB protein (Fig 3G). The seven glycan sites are conserved among different EBV strains (S3 Fig) but most of them are not present in other herpesviruses (S4 Fig). We next constructed seven *N*-glycosylation-site-defective mutants by substituting Asn with Gln, and named them N76Q, N163Q, N290Q, N348Q, N395Q, N436Q and N629Q. However, these single-site mutations did not cause appreciable changes in the molecular weight or Con A binding of gB (Fig 3H), nor did they impede its interaction with FBXO2 (S5A Fig), indicating that gB is glycosylated at multiple sites. To fully characterize the glycosylation sites, seven Asn residues were mutated to Gln sequentially from the N-terminus to the C-terminus of the gB ectodomain; these mutants were designated by the number of Asn residues mutated, from 1NQ to 7NQ (Fig 3F). The electrophoretic migration patterns of the single to seven point mutants showed that the migration rate gradually increased as more Asn residues were mutated; accordingly, their binding to Con A agarose was gradually reduced, suggesting that all seven Asn residues were glycosylated *in vivo* (Fig 3I). We next examined the interaction of FBXO2 with these glycosylation-defective mutants. Co-IP results suggested that mutation of the first two Asn residues (N76 and N163) began to reduce the gB-FBXO2 association, and mutations of three or more Asn residues largely abolished their interactions (Fig 3J and 3K), suggesting that FBXO2 association is dependent on the number of mannose moieties on gB. In summary, we identified the glycosylation sites on gB and revealed a glycosylation-dependent interaction between FBXO2 and gB.

### FBXO2 ubiquitinates and degrades glycosylated gB

**Fig 4:**
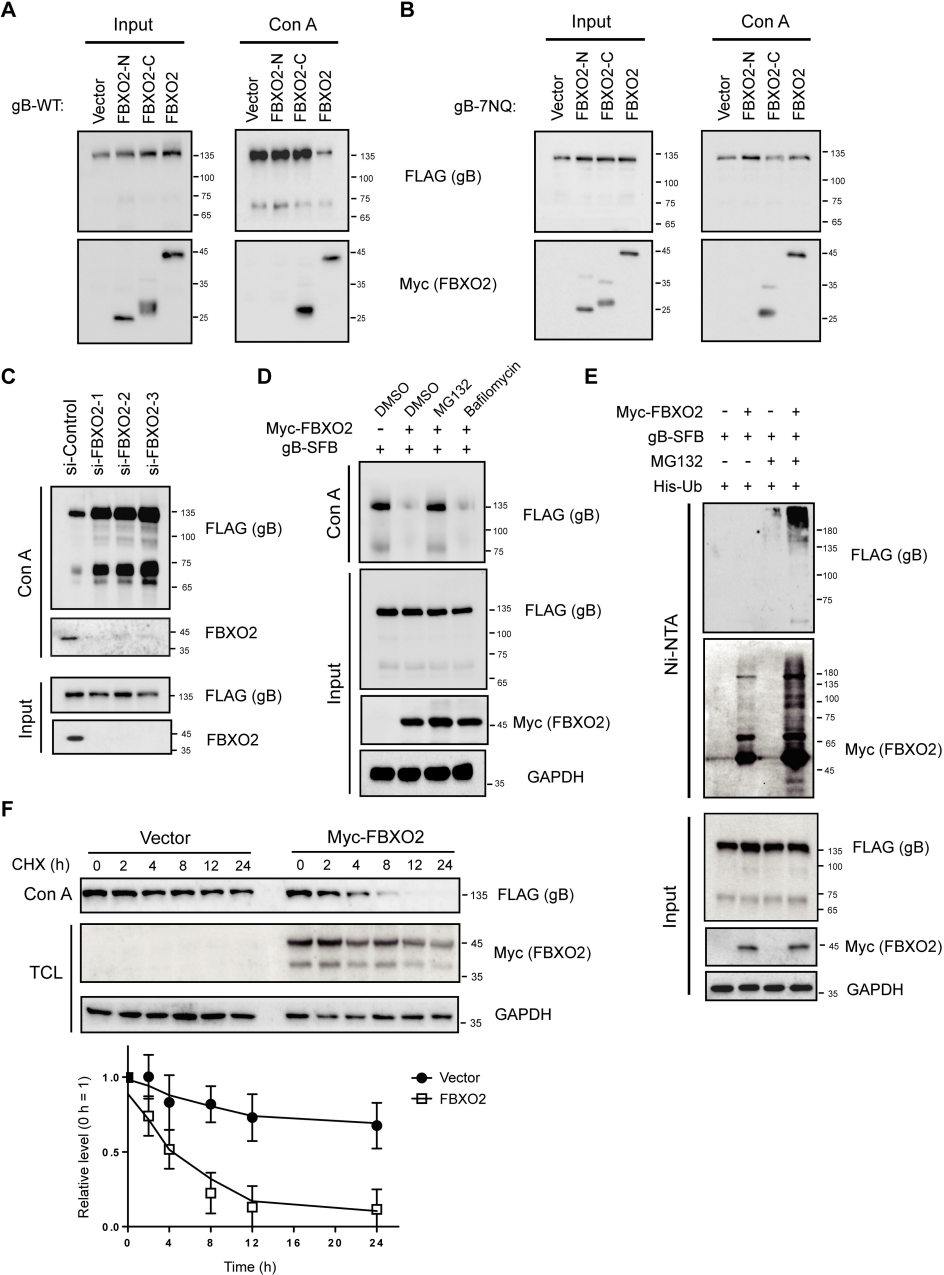
FBXO2 ubiquitinates and degrades *N*-linked glycosylated gB. **(A)** FBXO2 reduces the level of glycosylated gB. HEK293T cells were co-transfected with gB, along with the FL, N-terminal or C-terminal FBXO2 constructs. 48 hrs later, the glycosylated proteins were enriched by Con A agarose pull-down, and the bound proteins and total cell lysates were immunoblotted with anti-FLAG and anti-Myc antibodies. **(B)** FBXO2 did not affect the total or glycosylated levels of the glycosylation-defective 7NQ gB mutant. The experiments were conducted as in **(A)**. **(C)** CNE2 cells stably expressing gB were transfected with three siRNAs targeting FBXO2 or a scramble control siRNA, and 72 h later, the cells were harvested and subjected to Con A pull-down and WB. **(D)** Effects of MG132 and bafilomycin A1 on the levels of total and glycosylated gB. **(E)** FBXO2 ubiquitinates gB *in vivo*. HEK293T cells were transfected with His-ubiquitin (Ub), gB-SFB and Myc-FBXO2 or empty vector. The cells were treated with or without 20 μM MG132 for 6 h before harvest. 48 hrs post-transfection, the cells were lysed in 6 M guanidine-HCl buffer, the His-tagged ubiquitinated proteins were enriched by Ni-NTA (nickel-nitrilotriacetic acid) pull-down, and the bound proteins were eluted by SDS loading buffer and immunoblotted with antibodies as indicated. **(F)** HEK293T cells stably expressing gB were transfected with empty vector or Myc-FBXO2, and 36 h later the cells were treated with 50 μg/mL cycloheximide (CHX) to block protein synthesis and collected at the indicated time points. (top) Total cell lysates were analyzed by WB; (bottom) the degradation of gB was calculated by determining the relative quantification of gB from WB results. The mean value for vector-transfected cells at 0 h was normalized to a relative protein level of 100% (n = 3).

Given the known substrate specificity of FBXO2 for high-mannose-containing glycoproteins [26–28,30] and the high-mannose modification of gB, we hypothesized that gB is recognized and ubiquitinated by SCF^FBXO2^ for proteasome-mediated degradation. To test this idea, we examined the protein level of gB in the presence or absence of FBXO2. However, overexpression of FBXO2 did not affect the total level of gB (Fig 4A, left panel). Considering that only a portion of gB molecules with high-mannose modification associated with FBXO2 (Fig 3E), we examined the level of glycosylated gB when FBXO2 was overexpressed. Con A agarose pull-down showed a dramatic reduction in bound gB when FBXO2 was present, while the two truncation mutants of FBXO2 had no effect, although the C-terminal SBD of FBXO2 bound to Con A at a rate comparable to that of the full-length protein (Fig 4A, right panel). By contrast, FBXO2 did not suppress the association of the glycosylation-defective 7NQ gB mutant with Con A agarose, although 7NQ exhibited low affinity for Con A agarose (Fig 4B). Conversely, depleting FBXO2 with three independent siRNAs greatly increased the level of glycosylated gB (Fig 4C), indicating that FBXO2 specifically degrades glycosylated gB.

To gain insight into the mechanism of gB degradation, we treated cells stably expressing gB with the proteasome inhibitor MG132 or the lysosome inhibitor bafilomycin A1. Con A pull-down suggested that only proteasome inhibition prevents FBXO2-mediated degradation of glycosylated gB (Fig 4D). To further confirm the involvement of the ubiquitin-proteasome system (UPS) in FBXO2-mediated gB degradation, HEK293T cells were transfected with constructs encoding His_6_-tagged ubiquitin (Ub), FBXO2 and gB and subjected to an *in vivo* ubiquitination assay under denaturing conditions. As expected, FBXO2 overexpression resulted in the enrichment of a high-molecular-weight, ubiquitinated gB species by nickel column pull-down, and FBXO2 auto-ubiquitination was also readily detectable (Fig 4E), These data suggested that FBXO2 promotes gB ubiquitination *in vivo*. Furthermore, we examined the half-life of glycosylated gB in the presence or absence of FBXO2 using a cycloheximide (CHX) chase assay. Glycosylated gB enriched by Con A agarose was stable up to 12 hours, whereas FBXO2 overexpression reduced the half-life of glycosylated gB to approximately 4 hours (Fig 4F). These data indicate that FBXO2 targets glycosylated gB for ubiquitin-mediated degradation.

### Depletion of FBXO2 promotes gB localization on the cell surface, consequently facilitating cell-cell fusion and EBV infection

**Fig 5:**
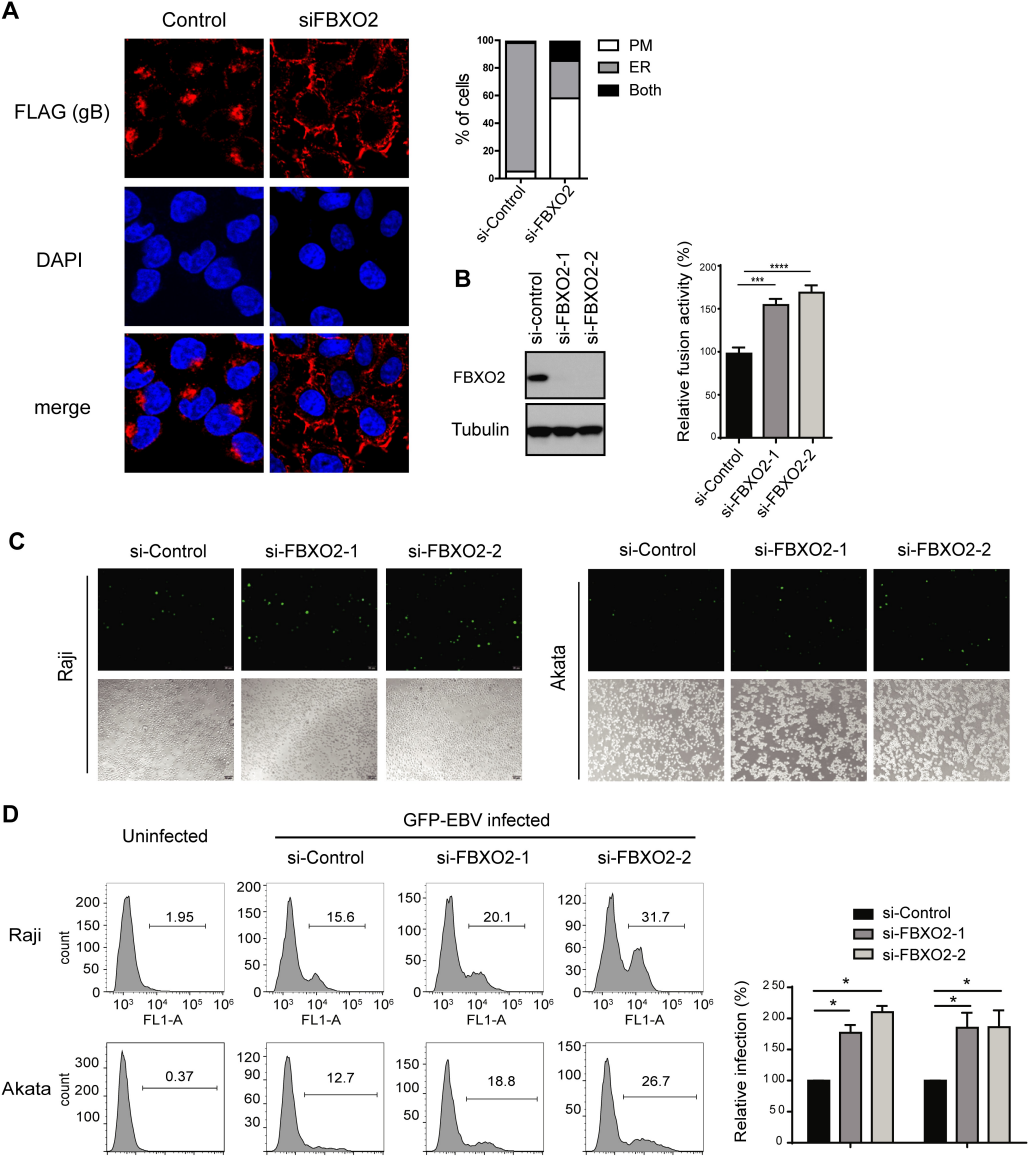
Glycosylation is required for gB-mediated cell fusion and EBV infection. **(A)** CNE2 cells that stably express SFB-tagged FBXO2 were transfected with control siRNA or siRNA targeting FBXO2. 72 hrs post-transfection the cells were fixed and subjected to immunofluorescence microscopy. (left) Immunofluorescence images representing the subcellular distribution of gB; (right) histogram representing the percentage of cells displaying the gB distribution on the ER, plasma membrane (PM) or both compartments. **(B)** Knockdown of FBXO2 promotes cell-cell fusion. The fusion assay was performed as described in “Methods”. The effector cells were co-transfected with control siRNA or siRNA targeting FBXO2. 48 hrs post-transfection, the effector and target cells were mixed. The relative fusion activity was calculated as the ratio of firefly to Renilla luciferase activity. The mean value for control siRNA-transfected cells was normalized to 100% relative fusion activity. n = 3, mean ± SD. **(C)** CNE2 EBV producer cells were transfected with control siRNA or siRNAs targeting FBXO2, and 60 h later, EBV lytic replication was induced by the addition of TPA and NaB for 12 h. The supernatants were collected and used to infect Raji (left) and EBV-negative Akata (right) cells. GFP-positive cells were indicative of successful infection. **(D)** (left) The EBV infection rate in B cells was determined by fluorescence-activated cell sorting (FACS) analysis based on GFP expression. EBV infection was performed as described in **C**, and the uninfected B cells were used as a negative control. (right) Quantification of GFP-positive cells. Data were represented as the mean ± SD of three independent experiments.

Having demonstrated that FBXO2 is the E3 ligase for EBV gB, we further investigated whether FBXO2 affects gB intracellular trafficking. Consistent with previous reports, EBV gB is retained in the ER and nuclear envelope and barely detectable on the plasma membrane (PM) [19,25] in both NPC and oral cancer cell lines (S5B and S5C Fig), while FBXO2 is a cytoplasmic protein (S5B and S5D Fig). Strikingly, upon depletion of FBXO2 by siRNA, the majority of gB translocated to the PM of cells (Fig 5A). The data indicate that loss of FBXO2 promotes gB translocation from the ER to the cell surface.

gB plays an essential role in virus entry by promoting cell-cell fusion. We took advantage of an established virus-free cell-based quantitative fusion assay [42] to determine the role of FBXO2 in gB fusogenic function. In accordance with the membrane transportation of gB by FBXO2 loss, depletion of FBXO2 increased the fusion activity of gB to 1.5-fold of that observed in cells transfected with control siRNA (Fig 5B). Next, we investigated the effect of FBXO2 on EBV entry. Using the established EBV producer cell line CNE2 carrying EBV-GFP, we produced EBV virions to infect the B cell lines Raji and Akata. Flow cytometry revealed that EBV-GFP virions originating from FBXO2-depleted CNE2 cells were more infectious than those originating from control cells (Fig 5C and 5D). To exclude the possibility that the enhanced infectivity induced by FBXO2 depletion was due to increased viral production, we examined lytic cycle induction and EBV copy number in EBV producer cells with or without FBXO2 knockdown, and the data suggested that FBXO2 had little effect on EBV production (S5E and S5F Fig). Collectively, these data suggest that loss of FBXO2 promotes gB maturation to enhance membrane fusion and viral entry.

## Discussion

In this study, we identified SCF^FBXO2^ as an E3 ubiquitin ligase targeting EBV envelope protein gB. In the SCF complex, adaptors are necessary to achieve substrate specificity. FBXO2 is unique among ~70 F-box proteins encoded by the human genome in its recognition for high-mannose glycans [26–30], and it has been reported to mediate the ubiquitination and degradation of various cellular glycoproteins [32–34,43–45]. High-mannose glycans contain unsubstituted terminal mannose sugars and have been regarded as incomplete products of the *N*-glycosylation pathway; they are evolutionarily older than complex *N*-glycans and are the typical oligosaccharides in yeast and other fungi. In mammalian cells, most glycans will be further modified in the Golgi by addition of various sugar residues, gaining complexity in sugar types and branching structure [41]. Structural studies of FBXO2 and glycan profiling indicate that FBXO2 binds Man_3_GlcNAc_2_, the common core pentasaccharide in *N*-linked glycans, and adding mannose residues did not affect the affinity [28–30]. Man_3_GlcNAc_2_ is the innermost moiety, which is difficult to reach in hybrid and complex *N*-glycans under native conditions; therefore, SCF^FBXO2^ is thought to degrade denatured *N*-linked glycoproteins nonspecifically. EBV gB is a unique viral glycoprotein in that its *N*-glycan modification is predominantly of the high-mannose type, regardless of the source of gB (virion or expressed in mammalian cells) [10,19,22,25]. All EBV gB glycans are sensitive to Endo H (Fig 3A). *N*-glycan MS analysis and mutagenesis studies identified seven glycan sites on gB, and mutation of each site led to similar small reductions in molecular weight; thus, each site has a set of homogeneous high-mannose-type oligosaccharides that exactly match the structural requirement of FBXO2. The exclusively mannose-terminated glycosylation pattern explains the substrate specificity of FBXO2 for gB, but not for gp350 and gH/gL (Fig 1C and S1 Fig), as gp350 and gH/gL harbor heterogeneous glycans [19], and sugar chains terminated with non-mannose glycans could not be reached by FBXO2 under native condition. Therefore, SCF^FBXO2^ acts as a specific E3 ligase for gB because of its unique sugar chain type.

High-mannose *N*-glycans are a signature of proteins in the ER. In accordance with its sugar chain type, EBV gB is primarily located at the ER [19,25]. We did observe a predominant ER localization of gB in epithelial cells (Fig 5A and S5B–S5D Fig); in contrast, depletion of FBXO2 substantially changes the subcellular distribution of gB from the ER to the PM (Fig 5A), suggesting that FBXO2 is a rate-limiting factor for the processing and maturation of gB in host epithelial cells. Because viral fusion proteins usually exist on the viral membrane surface to promote membrane merging, it is not surprising that the loss of FBXO2 promotes cell fusion and viral entry. Spatially, FBXO2 distributes at the cytoplasm, whereas gB normally resides in the ER as determined by immunofluorescence imaging and subcellular fractionation (S5B and S5D Fig). As a type I membrane protein, the N-terminal ectodomain of gB faces the lumen of the ER, where all the glycosylation residues located; moreover, the 26 S proteasomes are present in the cytoplasm but absent in organelles such as ER. In this regard, FBXO2 is highly likely to degrade gB via ER-associated degradation (ERAD), and the degradation of gB occurs in the ER via retrotranslocation. Taking together with the PM transportation of gB by depletion of FBXO2, it is conceivable that gB is retained in the ER and undergoes rapid equilibration between folding and unfolding, with the unfolded gB retrotransported to the cytoplasm to be degraded by SCF^FBXO2^. Depletion of FBXO2 shifts the equilibrium to refolding, and as a result, most gB is folded correctly and then translocated from the ER to the cell surface. Therefore, FBXO2 acts as a host restriction factor for gB maturation.

gB exists in almost all herpesviruses. gBs of other γ-herpesviruses, such as murine gamma herpesvirus 68 (MHV-68), have also been demonstrated to be high-mannose *N*-linked glycoproteins retained in the ER [46]. In addition, β-herpesvirus cytomegalovirus (HCMV) is also exclusively modified with high-mannose *N*-glycans [47]. By contrast, gB of α-herpesviruses, such as HSV-1 and HSV-2, are only partially susceptible to Endo H treatment and are transported through the Golgi to the membrane [17,18]. Sequence alignment indicates that although EBV gB shares homology with herpesvirus gB homologs, most glycan sites on EBV gB are not conserved in other herpesviruses; even in γ-herpesviruses, only two glycan sites (N348 and N629) are conserved (S4 Fig). It remains to be determined whether other β- and γ-herpesviruses gBs are also regulated by the SCF^FBXO2^ E3 ligase in their host cells.

We demonstrated that the degradation of gB by SCF^FBXO2^ in epithelial cells impedes entry of the progeny virus into B cells. However, as gB is more essential for epithelial cell penetration than for B cell entry [20], we could not exclude the possibility that FBXO2 also negatively regulates the spreading of EBV to surrounding epithelial cells. We are currently unable to address this issue because of the very low efficiency of EBV re-entry into epithelial cells, which is in part due to the high levels of gp42 on EBV virions originating in epithelial cells [48,49].

**Fig 6:**
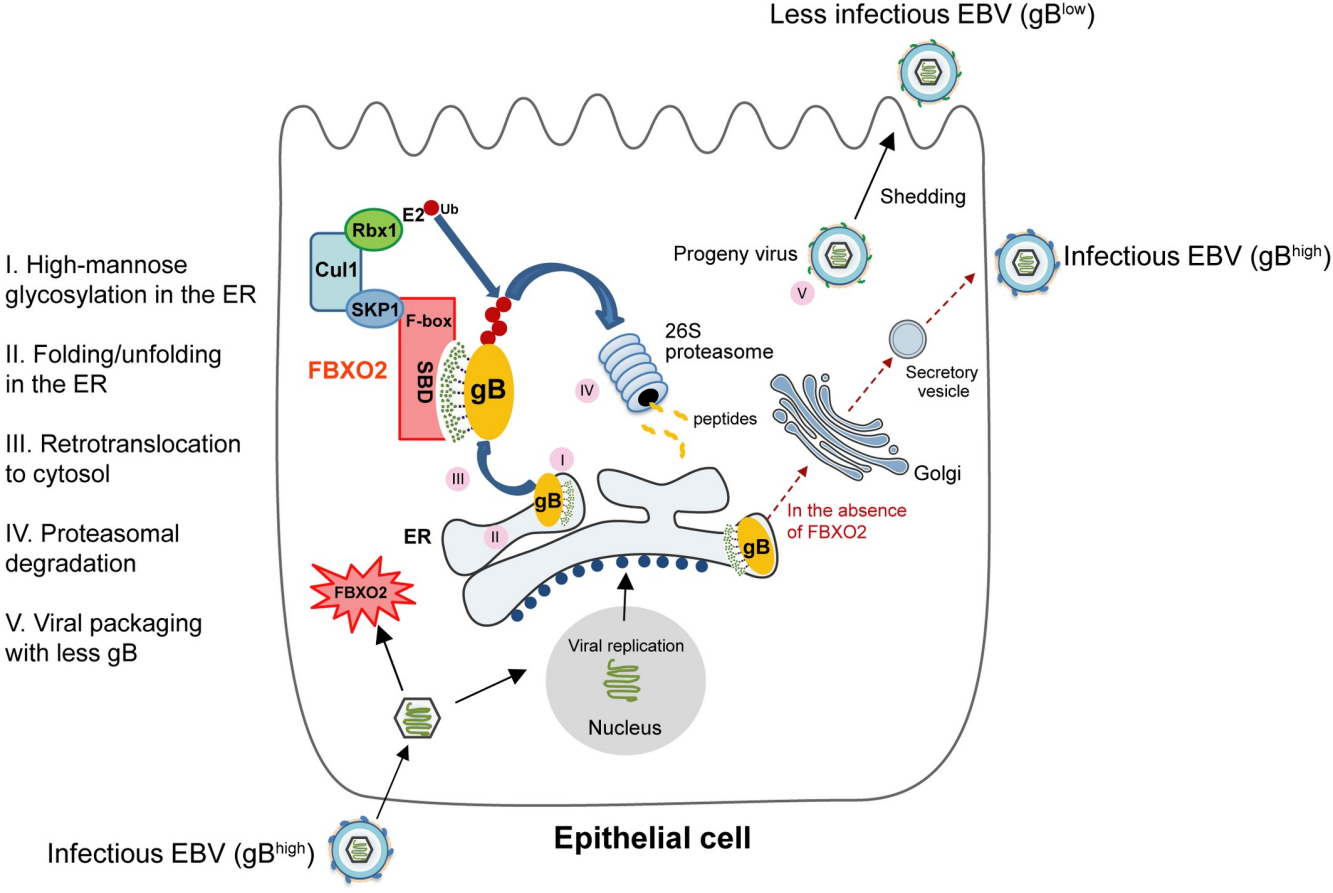
Working model for FBXO2-mediated glycosylation-dependent degradation of gB in epithelial cells.

The significance of epithelial-specific expression and EBV-induction of FBXO2 is an unanswered question. Similar to other envelope proteins, gB is only expressed in the lytic cycle to release infectious viral particles. EBV replicates poorly in B cells and only occasionally reactivates from latency; in contrast, human pharyngeal epithelial cells are believed to be reservoirs of lytic EBV, in which lytic replication directly follows viral entry. In this regard, anti-EBV lytic infection mechanisms appear to be more important for oral and nasopharyngeal epithelial cells than for B cells. We demonstrate that EBV infection stimulates FBXO2 expression in NPC cells, however the scenario did not happen in the BL cell lines and gastric carcinoma cell line AGS (Fig 2), the underlying mechanism of FBXO2 regulation by EBV is still elusive. In turn, FBXO2 degrades the newly synthesized gB, which forms a negative feedback loop to attenuate the infectivity of progeny viruses (Fig 6). To our knowledge, this is one of very few examples of how epithelial cells defend against EBV invasion. This finding might help gain a deeper understanding of the interplay between host and virus.

## Methods

### Cell culture and establishment of primary NPC cells and stable cell lines

Raji is an EBV-positive BL cell line, and Akata-EBV is an Akata BL cell line carrying the Akata bacterial artificial chromosome (BAC); both were cultured in RPMI 1640 medium with 10% fetal bovine serum (FBS). The NPC cell lines CNE1, CNE2, HNE1, HONE1, HK1, SUNE1 and C666-1, oral cancer cell lines SCC-15, SCC-25, UM1, TSCCa, Tca8113 and CAL 27, and gastric cancer cell lines AGS, AGS-EBV, MGC803 and MKN-45 were cultured in Dulbecco’s modified Eagle medium (Gibco) with 10% FBS. Normal oral keratinocyte NOK, immortalized nasopharyngeal epithelium cell lines NPEC2-Tert and NPEC5-Tert were cultured in keratinocyte serum-free growth medium supplemented with 5 μg/L EGF and 50 mg/L Bovine Pituitary Extract (Gibco). EBV-positive CNE2 and HNE1 cell lines are parental cell lines carrying Akata-EBV-GFP and were cultured in the presence of G418 (500 μg/mL). NOK and HEK293T were from ATCC; oral cancer cell lines, gastric cancer cell lines, NPE and NPC cell lines were provided by Prof. Mu-Sheng Zeng (Sun Yat-sen University Cancer Center); the BL cell lines were provided by Prof. Wenqi Jiang (Sun Yat-sen University Cancer Center); HEK293-M81 cell line carrying the EBV M81 strain was kindly provided by Prof. Dong-Yan Jin (The University of Hong Kong).

Two primary NPC cell lines were isolated from nasopharynx biopsy samples from two male Chinese NPC patients. Briefly, tumor samples were placed in HBSS buffer and minced with sterile scissors. The minced tumor samples were then digested with 1 mg/mL Collagenase I for 15 min at 37°C and cultured in keratinocyte serum-free growth medium supplemented with EGF and Bovine Pituitary Extract.

To generate stable gB-expressing cell lines, gB was cloned into the lentiviral vector pDEST-C-SFB and packaged into lentiviruses by co-transfection with the packaging plasmids pMD2G and pSPAX2 in HEK293T cells. Forty-eight hours after transfection, the supernatant was collected and used for infection. Stable pools were selected with medium containing 2 μg/mL puromycin.

### Constructs

All cDNAs encoding EBV proteins were amplified from the M81 bacmid. cDNA fragments were subcloned into pDONR201 (Invitrogen) entry vectors and subsequently transferred to gateway-compatible destination vectors. pDEST-C-SFB was used for C-terminal tagging with SFB (S-, Flag and streptavidin-binding tag; gift from Prof. Junjie Chen), and pDEST-Myc was used for N-terminal tagging with Myc. N-to-Q point mutants were generated by site-directed mutagenesis PCR with Primerstar GXL (Takara). FBXO2 was cloned into pGEX-4T1 for prokaryotic expression and the *in vitro* binding assay. All constructs were verified by sequencing.

### TAP, immunoprecipitation and pull-down assays

HEK293T, CNE2 and HNE1 cells stably expressing gB-SFB were amplified and subjected to TAP according to our previous methods[31]. MS analyses were performed by Beijing Proteome Research Center and Wininnovate Bio. (Shenzhen, China).

For immunoprecipitation, cells were lysed in NETN buffer (20 mM Tris-HCl at pH 8.0, 100 mM NaCl, 1 mM EDTA, 0.5% NP-40) containing 50 mM β-glycerophosphate, 1 μg/mL pepstatin A and 10 μM leupeptin, and the bait proteins were precipitated by S-protein beads (Navagen), Streptavidin beads (Amersham) or anti-c-Myc Tag affinity gel (Biolegend) as indicated.

For the GST pull-down assay, GST or GST-FBXO2 recombinant protein immobilized onto glutathione Sepharose 4B beads (GE Healthcare) was incubated with gB protein purified from stably infected HEK293T cells in NETN buffer for 4 h at 4°C. For Con A pull-down, cell lysates were incubated with Concanavalin A (Con A) agarose (Vector Labs) for 4 h at 4°C. The bound proteins were eluted with 1X SDS loading buffer.

### *N*-glycan profiling by shotgun glycomics analysis

HEK293T cells stably expressing SFB-tagged gB were used to purify the gB protein by streptavidin agarose pull-down followed by extensive washing with high-salt NETN buffer (containing 400 mM NaCl); gB was then eluted with 2 mg/mL biotin (Sigma). The eluted protein was dialyzed in PBS and concentrated by ultrafiltration (Amicon Ultra 10 MWCO) to 0.5 μg/μL. A total of 2.5 μg of gB protein was sent for MS/MS analysis by Shanghai Applied Protein Technology Co. Ltd. Briefly, the protein was reduced with 100 mM DTT, denatured in 8 M urea buffer and alkylated with iodoacetamide (IAA) to prevent disulfide bond formation. After a 30 min incubation in the dark at room temperature, the sample solution was diluted in 50 mM NH_4_HCO_3_ buffer containing 2 μg of trypsin, 2 μg of chymotrypsin and 2 μg of Glu C and digested at 37°C overnight. Tryptic peptides were digested with 1 μL of PNGase and dried by vacuum centrifugation. Nanoscale liquid chromatography coupled to tandem MS (nano LC-MS/MS) was performed by separating the peptide mixture using an Easy-nLC liquid chromatograph system with a Thermo Scientific EASY column (Thermo Fisher), followed by ESI MS identification using a Q-Exactive Hybrid Quadrupole-Orbitrap Mass Spectrometer (Thermo Finnigan). Raw data from the Q-Exactive were converted into MGF files via Proteome Discoverer 1.3 (PD1.3, Thermo). The subsequent searches were carried out using Mascot Daemon (version 2.2, Matrix Science) and filtered based on a peptide false discovery rate (FDR) ≤ 0.01%.

### Antibodies, chemicals and siRNAs

The following antibodies were used in this study: FBXO2 (sc-398111, Santa Cruz), EBNA1 (ab81581, Abcam), Zta (sc-53904, Santa Cruz), β-tubulin (sc-23948, Santa Cruz), Myc (sc-40, Santa Cruz), GAPDH (60004-1-1g, Protein-tech) and FLAG (F3165, Sigma). MG132, bafilomycin A1 and tunicamycin were purchased from Selleckchem, LLC. siRNAs were synthesized by Guangzhou RiboBio Co., Ltd. The siRNAs were 21 base pairs long, and the sequences were as follows: control siRNA: UUCAAUAAAUUCUUGAGGUdTdT; FBXO2 siRNAs: #1: GAUGAGAGCGUCAAGAAGUdTdT; #2: CAGUUCUACUUCCUGAGCAdTdT; #3: AAGGUAGAUAGGCCUUAACdTdT. Lipofectamine RNAiMAX (Invitrogen) was used for siRNA transfection.

### CRISPR/Cas9-mediated *EBNA1* deletion

CRISPR/Cas9-mediated EBV clearance in C666-1 cells was performed as previously reported [39]. Two gRNAs targeting *EBNA1*, 5’-GTGTGAATCATGTCTGACGA-3’ and 5’-GGCCCTGATCCTGAGCCGCC-3’ were cloned into the lentiviral transfer vector Lenti-CRISPRv2 and were used to package lentiviral particles. Stable *EBNA1*-deleted C666-1 cells were selected with 2 μg/mL puromycin, and the cells were used in experiments within one week after complete puromycin selection.

### RNA isolation and quantitative real-time PCR

Total RNA was extracted with Trizol reagent (Invitrogen). The EBV copy number was determined by real-time PCR analysis of the *BALF5* gene; the primer sequences were as follows: forward, 5’-GGTCACAATCTCCACGCTGA-3’; reverse, 5’-CAACGAGGCTGACCTGATCC-3’. The primers for *FBXO2* were as follows: forward, 5’- CCACGATGAGAGCGTCAAGA -3’; reverse, 5’- GAGCTCGTAGAGGCAACCAG-3’.

### Cell fusion assay, virus production and cell-free EBV infection

The cell fusion assay was performed as previously described [42,50]. Briefly, HEK293T cells transfected with plasmids encoding T7 polymerase, gB, gH and gL were used as effector cells; another group of HEK293T cells transfected with the reporter plasmid pT7EMCLuc encoding the luciferase gene driven by the T7 polymerase and an internal control plasmid pRL-SV40 encoding the *Renilla* luciferase gene driven by the SV40 promoter served as target cells. Twenty-four hours after transfection, the effector and target cells were trypsinized and co-cultured in a 24-well plate for an additional 24 h. Firefly and *Renilla* luciferase activities were assayed by using the dual-luciferase reporter assay system (Promega) with a Veritas luminometer (Promega). The ratio of firefly luciferase activity to *Renilla* luciferase activity was regarded as the relative fusion activity. The mean value of the control group was normalized to 100% relative fusion activity.

For EBV production, Akata-EBV-GFP cells were treated with 0.8% (v/v) goat anti-human IgG for 6 h, CNE2-EBV-GFP cells were treated with 20 ng/mL 12-o-tetradecanoylphorbol-13-acetate (TPA) and 2.5 mM sodium butyrate for 24 h to induce EBV-infected cells to transition from the latent phase into the lytic cycle. After culture in fresh medium for 3 days, the supernatants were collected for infection. To infect B cells, 1 mL of 10 mL of supernatant collected from a 10 cm dish of CNE2-EBV-GFP cells was added to 1 × 10^5^ Raji cells or EBV-negative Akata cells for 2 h at 37°C. The cells were then washed with PBS and incubated for a further 36 h in fresh medium. The EBV infection rate was determined by calculating the percentage of GFP-positive cells using flow cytometry (Beckman) results analyzed by FlowJo software.

**Immunofluorescence, cycloheximide chase and *in vivo* ubiquitination assay** have been described previously [51].

**Xenograft and immunohistochemistry (IHC)** Xenograft and IHC were carried out as described previously [52]. For xenograft studies, female BALB/c nude mice (6 weeks old) were purchased from Shanghai Laboratory Animal Center.

### Ethics statement

All mouse experiments were performed in strict accordance with all provisions of the Animal Welfare Act, the Guide for the Care and Use of Laboratory Animals, and the PHS Policy on Humane Care and Use of Laboratory Animals. The protocol was approved by the Institutional Animal Care and Use Committee of Sun Yat-sen University (Protocol no. GZR2016-105). Primary NPC cell lines were collected from two adult patients with newly diagnosed NPC at the Sun Yat-sen University Cancer Center in 2017. Written informed consent was provided by all patients before the tumor biopsies were obtained. This study was conducted under the provisions of the Declaration of Helsinki and approved by Ethics Committee of Sun Yat-sen University Cancer Center (Protocol No. YB2013-04).

### Deglycosylation experiment

Recombinant gB protein was purified from HEK293T cells stably expressing gB-SFB by streptavidin bead pull-down followed by biotin (2 mg/mL) elution. Deglycosylation of gB with Endo H, PNGase F or Protein Deglycosylation Mix (all from NEB) was performed according to the manufacturer’s recommendations. Enzyme digestion was performed for 3 h at 37°C.

### Statistical analysis

Statistical analyses were performed with GraphPad PRISM software (GraphPad Software Inc., San Diego, CA). The IHC values for FBXO2 in tissues from the mouse tumorigenesis model were calculated using Image-Pro Plus 6.0.

## Supporting information

S1 Table TAP-MS list of gB-interacting proteins in HEK293T cells. (XLSX)

S2 Table TAP-MS list of gB-interacting proteins in CNE2 cells. (CSV)

S3 Table TAP-MS list of gB-interacting proteins in HK1 cells. (CSV)

S1 Fig TAP-MS data of other EBV glycoproteins and their association with FBXO2. **(A)** List of the known receptors for the EBV glycoproteins identified by TAP-MS. TAPs were carried out in HEK293T cells stably expressing SFB-tagged gp350, gp42 and gH/gL. **(B)** Co-IP of SFB-tagged gH/gL or gB with Myc-tagged FBXO2. The cell lysates were subjected to immunoprecipitation by anti-Myc agarose. **(C)** Co-IP of SFB-tagged gp350 or gB with Myc-tagged FBXO2. The experiments were carried out as described in **(B)**.
(JPG)

S2 Fig Identification of *N*-glycosylation sites on gB and the interaction between gB single glycan site mutants and FBXO2. ESI mass spectra of the *N*-glycans on positions N76, N290, N348, N395 and N436 of EBV gB.
(JPG)

S3 Fig Alignment of EBV gB protein from strains including M81, GD1, Akata and B95.8. Glycosylation sites are indicated with red boxes.
(JPG)

S4 Fig Comparison of gB homologs from representative herpesviruses in relation to EBV gB. (top) Homology tree of herpes virus gB. (bottom) Multiple sequence alignment of gB homologs. A protein alignment of gB homologs from EBV (strain B95-8), HSV-1 (strain KOS), HSV-2 (strain HG52), Kaposi’s sarcoma-associated herpesvirus (isolate GK18), CMV (strain AD169), varicella-zoster virus (strain Oka vaccine) and Murid herpesvirus 4 was performed using Clustal Omega. Their UniProtKB accession numbers are indicated. Seven glycosylation sites are indicated with red boxes. The other two predicated glycosylation sites that were not included in this study are shown in green boxes.
(JPG)

S5 Fig Subcellular distribution of gB and FBXO2. **(A)** Co-IP experiments performed in HEK293T cells co-transfected with Myc-FBXO2 and SFB-tagged single glycosylation site mutants of gB. Cell lysates were immunoprecipitated by anti-Myc agarose (top) or S-protein beads (bottom) and immunoblotted with anti-Myc and anti-FLAG antibodies. **(B)** Immunofluoresence images of FLAG-tagged gB and Myc-tagged FBXO2 in the co-transfected CNE2 cells. **(C)** Oral cancer cell line Tca8113 was transfected with gB-SFB and processed for immunofluoresence staining. **(D)** Subcellular fractionation of CNE2 cells that stably expressing SFB-tagged gB. Cells were sequentially extracted by buffers containing 25 μg/ml digitonin followed by 1% NP-40. Digitonin only permeabilizes plasma membrane but not organelle membrane, while NP-40 solubilizes endomembranes. Each extract was analyzed by immunoblotting by antibodies as indicated. GAPDH stands for the cytosolic proteins existed in the digitonin extract (lane I: cytoplasma), and the Golgi protein GM130 represents organelle proteins present in the NP-40 extract (lane II: organelles). **(E)** Induction of EBV lytic replication in CNE2-EBV producer cells transfected with control siRNA or siRNAs targeting FBXO2. The switch to the EBV lytic cycle was indicated by GFP expression driven by a lytic promoter. **(F)** Relative EBV copy number in supernatants from control or FBXO2-knockdown CNE2-EBV producer cells as determined by real-time PCR using primers directed to the *BALF5* gene.
(JPG)
