# Supplementary figures and images for: Epstein-Barr virus activates F-box protein FBXO2 to limit viral infectivity by targeting glycoprotein B for degradation
Source: PLoS Pathog. 2018 Jul 27;14(7):e1007208. doi: 10.1371/journal.ppat.1007208 (PMC6082576; doi:10.1371/journal.ppat.1007208)

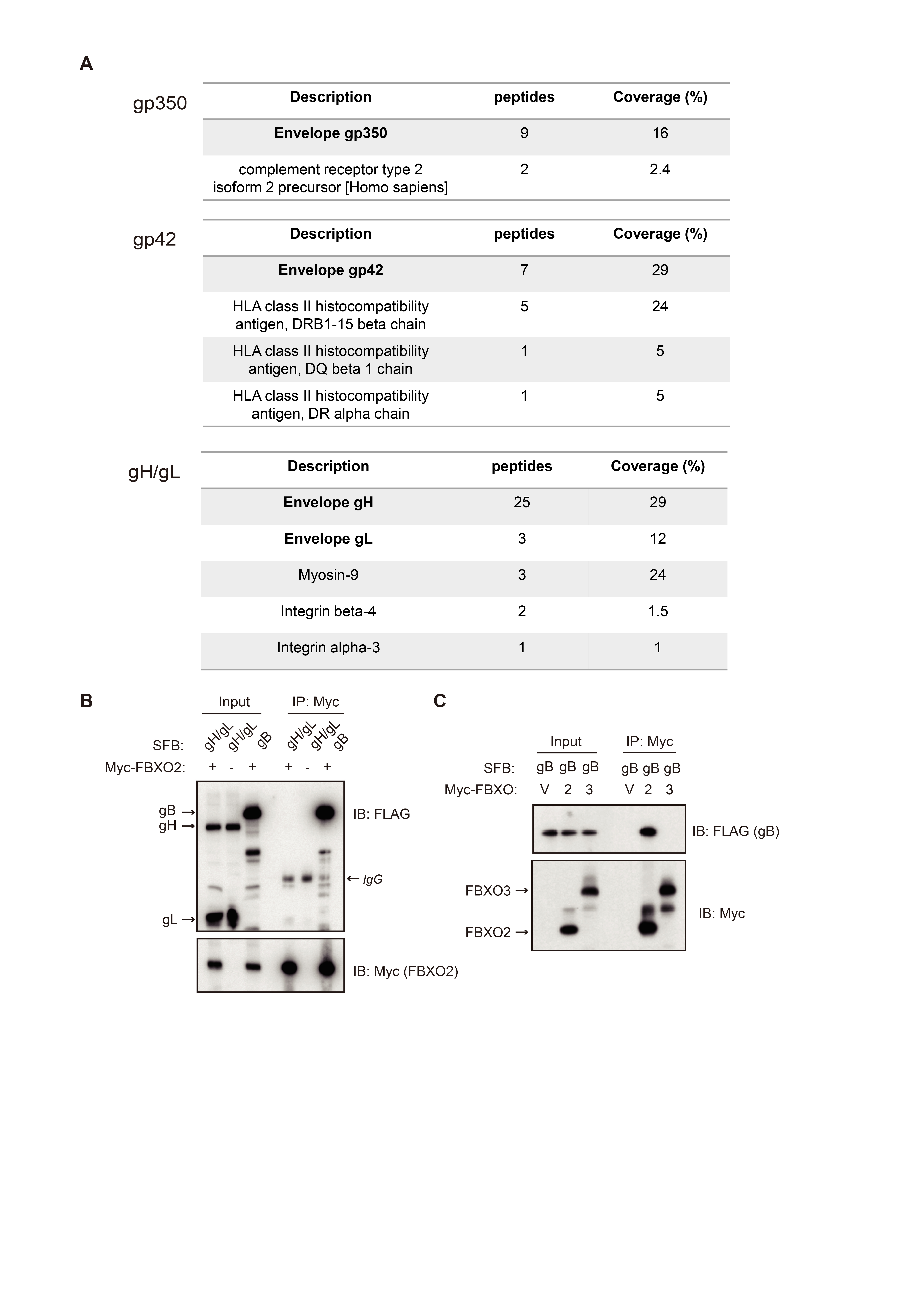

Supplement: S1 Fig — (A) List of the known receptors for the EBV glycoproteins identified by TAP-MS. TAPs were carried out in HEK293T cells stably expressing SFB-tagged gp350, gp42 and gH/gL. (B) Co-IP of SFB-tagged gH/gL or gB with Myc-tagged FBXO2. The cell lysates were subjected to immunoprecipitation by anti-Myc agarose. (C) Co-IP of SFB-tagged gp350 or gB with Myc-tagged FBXO2. The experiments were carried out as described in (B). (JPG) [file ppat.1007208.s004.jpg]

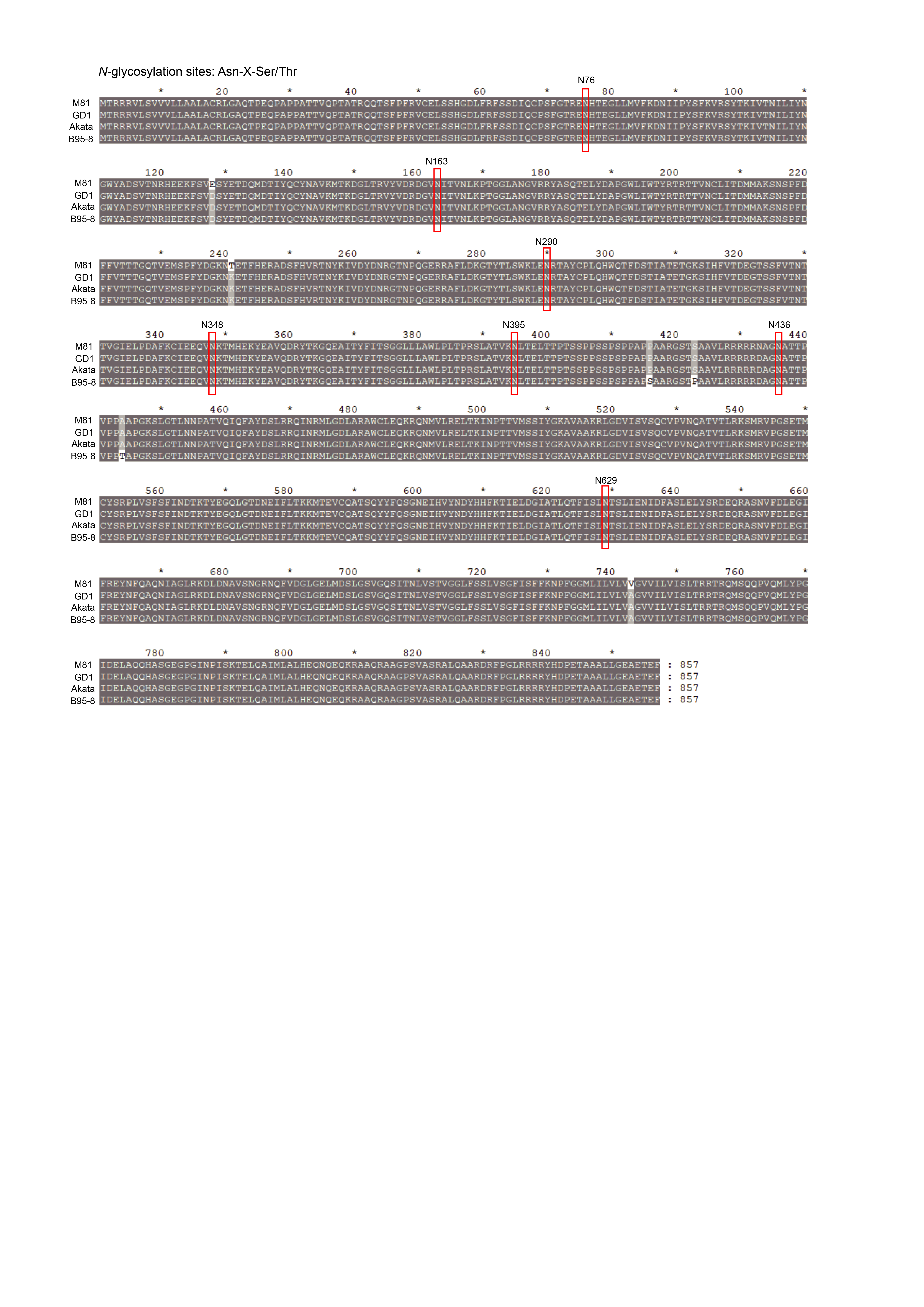

Supplement: S3 Fig — Glycosylation sites are indicated with red boxes. (JPG) [file ppat.1007208.s006.jpg]

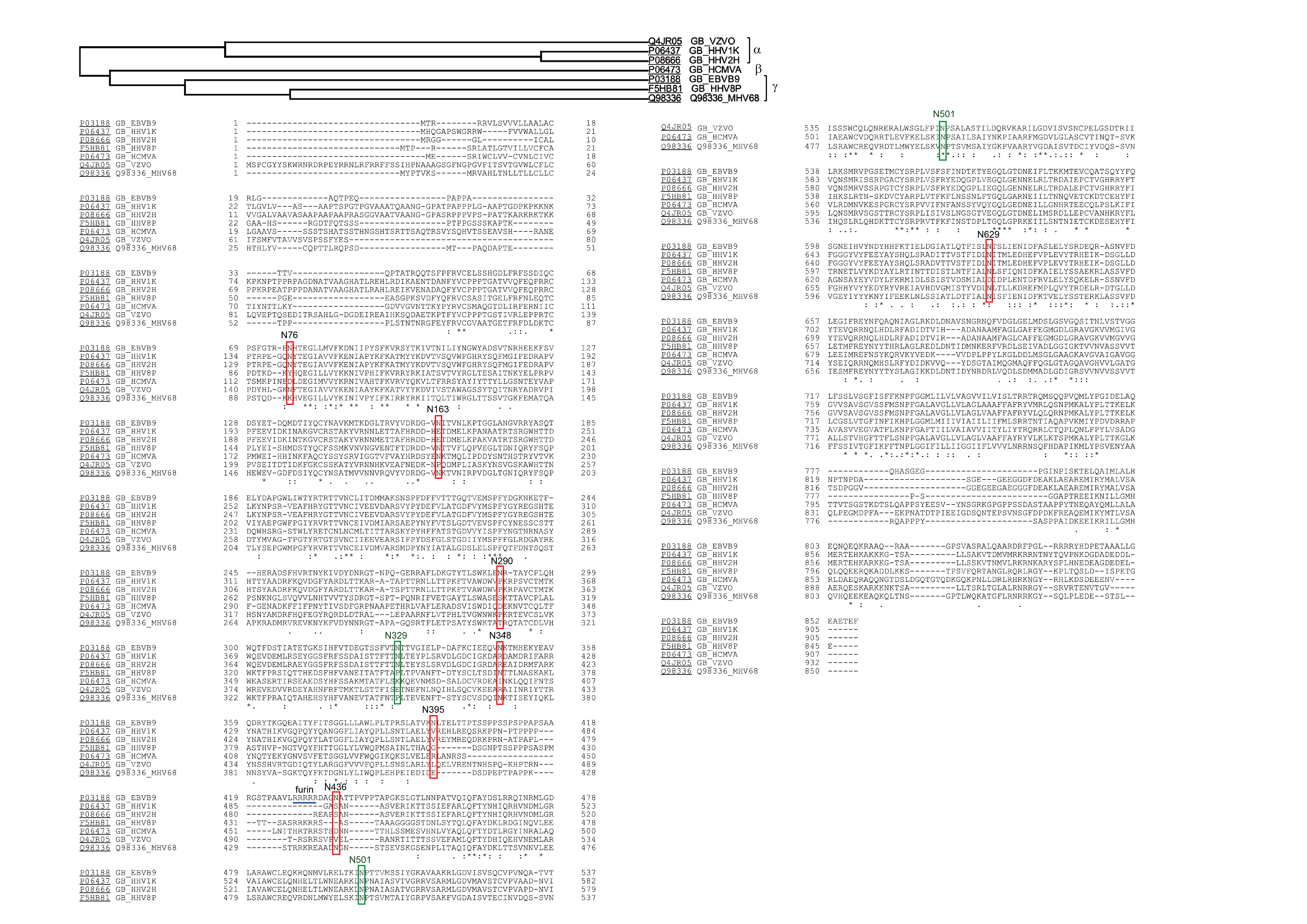

Supplement: S4 Fig — (top) Homology tree of herpes virus gB. (bottom) Multiple sequence alignment of gB homologs. A protein alignment of gB homologs from EBV (strain B95-8), HSV-1 (strain KOS), HSV-2 (strain HG52), Kaposi’s sarcoma-associated herpesvirus (isolate GK18), CMV (strain AD169), varicella-zoster virus (strain Oka vaccine) and Murid herpesvirus 4 was performed using Clustal Omega. Their UniProtKB accession numbers are indicated. Seven glycosylation sites are indicated with red boxes. The other two predicated glycosylation sites that were not included in this study are shown in green boxes. (JPG) [file ppat.1007208.s007.jpg]
